# Innovative Approaches to Early Detection of Cancer-Transforming Screening for Breast, Lung, and Hard-to-Screen Cancers

**DOI:** 10.3390/cancers17111867

**Published:** 2025-06-02

**Authors:** Shlomi Madar, Reef Einoch Amor, Sharon Furman-Assaf, Eitan Friedman

**Affiliations:** 1 SpotitEarly Inc., Englewood, NJ 07631, USA; 2 SpotitEarly Ltd., Kibbutz Hama’apil 3885700, Israel; reef@spotitearly.com; 3 Medical Writer and Consultant (Independent), Tel Aviv 6971028, Israel; sharon.furman@gmail.com; 4 Assuta Medical Centers, Tel-Aviv 6971028, Israel; feitan@tauex.tau.ac.il; 5 Gray Faculty of Medical and Health Sciences, Tel-Aviv University, Tel-Aviv 6997801, Israel

**Keywords:** early detection, liquid biopsy, non-invasive techniques, surveillance scheme, volatile organic compounds

## Abstract

Early detection of cancer is crucial for improving patient outcomes. Traditional modalities such as mammography and low-dose computed tomography are effective but exhibit inherent limitations, including radiation exposure and accessibility challenges. This review explores innovative, non-invasive cancer screening methods, focusing on liquid biopsy and volatile organic compound (VOC)-based detection platforms. Liquid biopsy analyzes circulating tumor DNA and other biomarkers in bodily fluids, offering potential for early detection and monitoring of treatment response. VOC-based detection leverages unique metabolic signatures emitted by cancer cells, detectable in exhaled breath or other bodily emissions, providing a rapid and patient-friendly screening option. We provide a comprehensive overview of these advanced multi-cancer detection techniques to enhance diagnostic accuracy, accessibility, and patient adherence, and ultimately enhance survival rates and patient outcomes.

## Simple Summary

Detecting cancer early can greatly improve a person’s chances of successful treatment. However, current screening methods have several drawbacks as they can be expensive, hard to access, and usually only check for one type of cancer at a time. Additionally, some of the deadliest cancers, such as pancreatic and ovarian cancer, still lack methods to identify them early. New non-invasive technologies, such as blood tests (liquid biopsies) and tests that analyze body scents based on volatile organic compounds (VOC), are showing promise. This review explores how these new methods, particularly those involving animal-assisted VOC detection and artificial intelligence, can be integrated into a more accessible and effective way to screen for several cancers at once. While challenges remain, ongoing research continues to move us closer toward a reliable system for early detection of many types of cancers.

## 1. Introduction

Cancer is a leading cause of morbidity and mortality around the world. In 2022, an estimated 20 million new cancer cases were identified, and 9.7 million cancer deaths occurred [1]. Early cancer-detection schemes play a crucial role in identifying cancer at its initial, potentially treatable stages before it spreads locally or to distant organs. These schemes have proven highly effective in reducing mortality rates across numerous cancer types [2]. Notable examples include a reduction in breast cancer (BC) and lung cancer (LC) mortality rates. LC was the most frequently diagnosed cancer worldwide, with over 2.4 million newly diagnosed cases in 2022. LC was also the primary cause of cancer-related deaths (over 1.8 million) in the same year [1]. BC is the leading cancer diagnosis and cause of cancer-related mortality in females. Globally, 2.3 million new cases and 670,000 deaths from female BC occurred in 2022, with geographic variation in age-standardized incidence rate and age-standardized mortality rate [1]. Recommended surveillance schemes for BC and LC include annual mammography for all women aged 45–74 years [3] and low-dose computed tomography (LDCT) for high-risk individuals (i.e., heavy smokers or those exposed to asbestos) from age 50 years [4], respectively. These surveillance schemes have been adopted by national professional bodies (e.g., the National Comprehensive Cancer Network [NCCN], https://www.nccn.org/guidelines/category_2, accessed on 12 March 2025), and the UK National Screening Committee ([UKNSC], https://www.gov.uk/government/organisations/uk-national-screening-committee accessed on 12 March 2025), and are routinely revisited by independent bodies such as the US Preventive Services Task Force (USPSTF-https://www.uspreventiveservicestaskforce.org/uspstf/ accessed on 12 March 2025). Yet, despite their proven mortality-lowering effect, these surveillance schemes have drawbacks and caveats, including radiation exposure, false-positive results, incidental findings, accessibility issues, and low compliance due to distrust in the healthcare system, augmented by the COVID-19 pandemic and disinformation campaigns. These and other challenges may underlie the low rate of acceptance to LC screening in the USA, estimated at 18.1% of eligible individuals in 2022, with the lowest rates (6.7%) noted in the 50–54 age group [5]. Moreover, according to the American Lung Association, only 5.8% of high-risk individuals were screened for LC [6]. The adherence rates to the recommended early-detection scheme for BC in the USA in 2021 (mammography every 2 years for average-risk women aged 50–74 years) have been higher at 75.9% [7]. However, these rates do not reflect the new USPSTF recommendation to lower the age of mammography screening to age 40 [8], which coincides with a recent report showing an increase in BC incidence rates among young American women [9]. Interestingly, between 2015 and 2019, LC incidence rates remained higher among women than men in the 35–54 age group [10].

Unlike the proven efficacy of early-detection schemes for BC and LC, there are cancer types for which no effective surveillance is currently available. Notable examples include pancreatic ductal adenocarcinoma (PDAC) and ovarian cancer (OvC). These two cancers are relatively rare. PDAC accounts for almost 90% of all diagnosed pancreatic tumors with global variations across age, race, and ethnicity [11]. In 2022, there were 510,992 new cases of PDAC diagnosed globally, with 60,127 new cases in the USA, with an age-standardized rate (ASR) of 4.7/100,000 and 8.6/100,000, respectively [12]. OvC ranked as the 10^th^-most common cancer among women in 2022, with 324,603 new cases. However, it is the fifth-leading cause of cancer-related deaths in females; 206,956 deaths from OvC occurred in 2022 [1,13], Geographic and ethnic variations across age-standardized incidence rates and histological subtypes were observed for ovC [14]. In addition to their relative rarity, the vague and non-specific nature of early-stage cancer symptoms, and the anatomical location of both organs, have all contributed to the lack of any clinically proven effective early-detection schemes for these cancer types, despite the ongoing search for serum biomarkers, targeted imaging modalities, and other means that have been tested but failed to show any clinically relevant effect. Early detection of these cancers has a transformative potential to improve survival outcomes. Survival rates in early-stage PDAC are significantly higher than those diagnosed at advanced stages. Localized pancreatic cancer has a 5-year relative survival rate of 44%, compared to 17% for regional disease and 3% for metastatic disease [15]. Stage I OvC (i.e., the disease is confined to the ovaries) has a 5-year survival rate of 89%, and stage II OvC (i.e., the disease is confined to the pelvis) is associated with a 5-year survival of 70%. However, stage III and IV disease have a 5-year survival rate of 40% and 20% or less, respectively [16].

Over the past 2 decades, there has been renewed interest in early detection of cancer as part of the preventive personalized approach to healthcare (e.g., [17]). These advancements include various liquid biopsy (LB) technologies [18,19,20], the development of novel multiomics biomarkers [21], technologies for detecting volatile organic compounds (VOCs) [22], and other biomarkers including biomineral deposits, such as psammoma bodies and microcalcifications [23,24,25,26]. In breast cancer, mammography-detected microcalcifications remain one of the earliest signs of malignancy [24]. Similarly, fine punctate calcifications on thyroid ultrasound strongly correlate with papillary thyroid carcinoma, showing high specificity for malignancy [25]. Psammoma bodies in cytologic or histologic specimens are characteristic of papillary tumors (e.g., ovarian serous and thyroid carcinoma) and may aid in early diagnosis [26]. In lung nodules, calcifications usually indicate benign lesions, though certain patterns can signify early malignancy [23]. These mineral biomarkers improve diagnostic sensitivity and could augment early-detection strategies.

**Figure 1:**
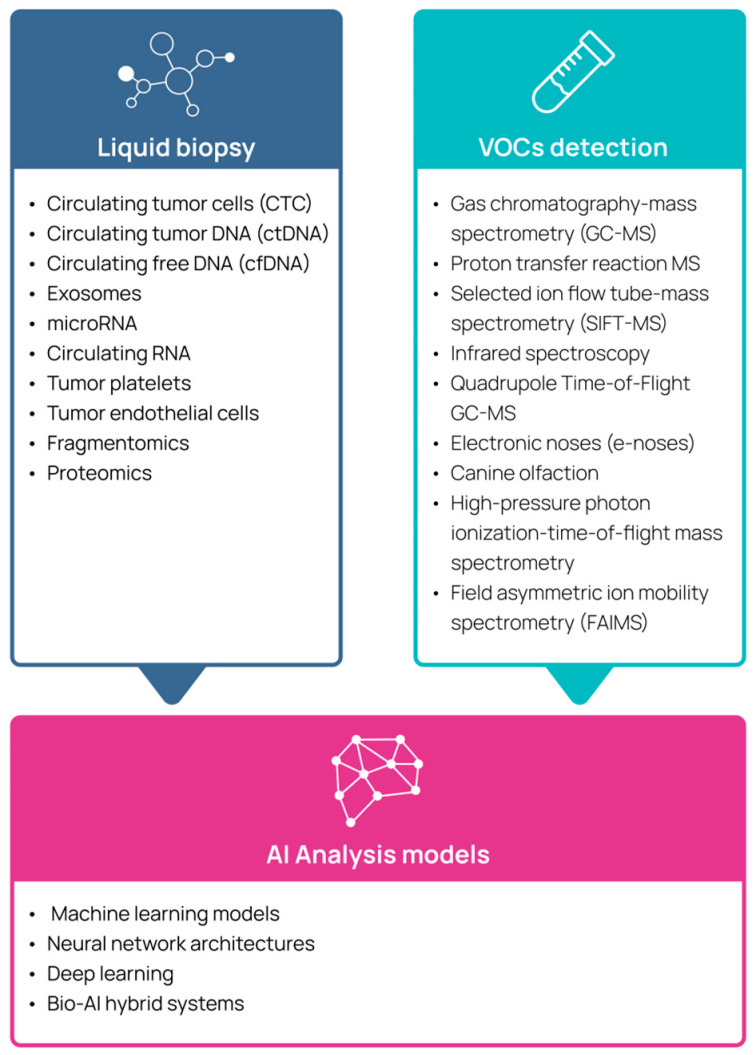
Emerging technologies used for multi-cancer detection.

Together, these innovations, which are illustrated in Figure 1 and are further described below, aim to enhance multi-cancer early detection (MCED) by improving existing diagnostic methods and expanding early detection opportunities to cancers that currently lack effective screening strategies, ultimately aiming to reduce overall and cancer-specific mortality.

## 2. Liquid Biopsy

LB refers to the sampling and analysis of tumor-derived material from body fluids, offering a non-invasive alternative to surgical tissue biopsies. In practice, a LB usually involves a simple blood draw, from which various circulating biomarkers can be isolated and studied. LBs encompass several components: circulating tumor cells (CTC), circulating tumor DNA (ctDNA), circulating free DNA (cfDNA), exosomes, microRNA, circulating RNA, tumor platelets, and tumor endothelial cells [27,28,29]. Since somatic, genetic, and epigenetic alterations, and their protein products, are the hallmark of cancer tissue, and the rate of cellular turnover in cancerous lesions is rapid, levels of cfDNA and their genetic/epigenetic composition reflect the somatic tumor landscape. A variety of assays utilizing cfDNA analysis have been reported as tools in early cancer detection, predominantly including either cfDNA multigene panel mutation analysis or cancer-specific methylation patterns [30]. LB offers several advantages over early-detection techniques currently in use: it is less invasive, offers faster turnaround time, may detect more than one tumor type in a single assay, and can capture tumor heterogeneity. Clinical studies reviewed over the past 2 years (e.g., [20]) have focused on evaluating the effectiveness of various LB techniques in detecting cancer at an early stage. For example, the PATHFINDER study, which evaluated a methylation-based MCED assay as a potential screening tool in individuals aged 50 years or older with no signs or symptoms of cancer, reported a 99.1% specificity. However, 57 out of 92 individuals who received a positive cancer signal were later determined to be false positives after a comprehensive diagnostic workup [31]. A separate study evaluating the same MCED test in 5461 asymptomatic individuals detected a cancer signal in 323 participants, with 244 ultimately diagnosed with cancer after a comprehensive workup. The test demonstrated an overall specificity of 98.4% and a sensitivity of 66.3%, which was lower for early-stage disease (24.2%) compared to later-stage disease (95.3%) [32].

LBs have gained substantial attention in cancers for which effective early-detection methods are currently unavailable, such as PDAC and OvC. For example, Cohen et al. [33] reported a 30% sensitivity in detecting PDAC among 221 cases compared to 182 controls using *KRAS* detection as a single biomarker. The sensitivity increased to 64% with a specificity of 99.5% when *KRAS* mutation detection was combined with four protein biomarkers (CA 19-9, carcinoembryonic antigen, hepatocyte growth factor, and osteopontin). Reese et al. [34] emphasized the need for a multi-biomarker approach for a more effective early detection of PDAC compared to detection with single biomarkers. A meta-analysis of 14 studies examining multigene-based LB techniques, encompassing 369 patients with PDAC (57% stage I-II), reported an overall pooled sensitivity and specificity of 70% and 86%, respectively. Interestingly, the concordance rate of detected mutations was 31.9% [35]. A LB analysis of CA19-9, ctDNA, and CTCs in 68 patients with pancreatic tumors (58 PDAC, 10 benign) showed sensitivity and specificity of 78% and 91%, respectively [36]. Most recently, Montoya–Mira et al. [37] described a novel assay that detects cancer-associated serum protease activity in plasma. When combined with the clinical biomarker CA 19-9, the assay achieved sensitivity and specificity of 85% and 96%, respectively, in detecting stage I PDAC, albeit with a limited sample size.

The use of LB for early detection and management of OvC has been the focus of multiple studies over the past 2 decades, and several reviews on the efficacy and the clinical use of different techniques and methodologies have been published (e.g., [38,39]). A review of studies published between 2009 and 2020 examined the use of various LB assays for the early detection of OvC. These studies encompassed 21–216 OvC cases across all disease stages, with reported sensitivities ranging from 23–98% and specificities between 56–100% [40]. Asante et al. [41] reviewed studies from 2012 to 2019 that evaluated the ability to detect high-grade serous OvC early. These studies, which analyzed 3–97 cases, employed mutation analyses of either a single gene (*TP53*) or up to 500 genes, with sensitivities ranging from 27–97% and specificities between 60–100%. More recently, Kuo et al. [42] reported that by setting a threshold of 4.75 flow cytometry-enriched CTC/mL, they could distinguish between benign (n = 9) and malignant (n = 26) ovarian lesions. The Canadian consortium CHARM is now evaluating a variety of LB technologies as an additional tool for surveillance in genetically predisposed individuals [43].

Despite the clear potential of LB as a clinical tool for early cancer detection, several challenges must be addressed before it can be widely incorporated into clinical practice. Key issues include standardization of methodologies, large-scale longitudinal validation studies, cost-effectiveness analyses, and the integration of artificial intelligence (AI) and novel imaging modalities [18,19].

## 3. Volatile Organic Compounds

### 3.1. The Scientific Basis for the Use of Volatile Organic Compounds in Cancer Detection

VOCs are a diverse group of carbon-based molecules that are metabolic byproducts of cellular processes. They are released into the circulatory system and can be detected in various body fluids, as well as exhaled air. More than 2000 VOCs have been identified in different body fluids [44]. Since cancer cells exhibit altered metabolism compared to non-cancerous tissue, distinct VOC profiles associated with specific cancers could serve as potential biomarkers for cancer detection [45,46].

Research on cancer-related VOCs has involved collecting samples from various biological matrices, including body fluids and excretions such as blood, urine, saliva, sweat, and feces, as well as exhaled air [45,47,48,49,50,51,52]. Several studies have reported the feasibility and effectiveness of VOC analysis in detecting multiple cancer types, including BC, colon cancer, prostate cancer, gastric cancer, and melanoma [45].

VOCs can be detected using analytical instruments such as gas chromatography coupled with mass spectrometry (GC-MS), proton transfer reaction mass spectrometry, selected ion flow tube-mass spectrometry (SIFT-MS), infrared spectroscopy, or *Quadrupole* Time-of-Flight GC-MS (GC-MS QTOF) [53,54]. Yet, the clinical use of GS-MS and its associated technologies is expensive, time-consuming, and requires highly trained and skilled personnel, making it labor intensive for routine medical practice. As a result, its widespread clinical application is unlikely, remaining largely confined to highly specialized laboratory environments [54].

### 3.2. Use of Electronic Noses for Volatile Organic Compound Detection

Electronic noses (e-noses), which mimic the olfactory system, are sensor arrays designed to detect and differentiate between various odor compounds, offering a cheap, fast, portable, and user-friendly approach for VOC analyses. Research on the use of e-noses in oncology has increased significantly and has shown promise in identifying specific VOC patterns associated with LC [22,55,56]. Use of machine-based VOC detection, such as e-noses, has some inherent limitations, such as low sensitivity and/or specificity, as well as poor reproducibility [57]. In a systematic review of the performance of e-noses used for cancer detection among 3677 individuals in 52 studies, the sensitivity of e-noses ranged from 48.3% to 95.8%, and the specificity ranged from 10% to 100% [58]. VOC profiles are also affected by factors unrelated to cancer, such as genetics, sex, diet, age, and the environment. Moreover, there is a lack of universal thresholds for “a cancerous VOC profile”, and hitherto, a scarcity of real-world clinical data supporting the clinical utility of e-noses.

### 3.3. Use of Canines for Volatile Organic Compound Detection

An alternative method for detecting VOCs with artificial means is the use of canines [59]. Canines have up to 220–300 million olfactory receptors in their noses, compared to about 5–6 million in humans [60,61], and can detect odors at concentrations of parts per trillion [62]. These features contribute to their exceptional scent detection capability. Furthermore, their olfactory bulb, which is devoted to analyzing smells, is proportionally 40 times larger than that of humans, allowing dogs to maintain a heightened focus on olfactory stimuli. The structure of the canine’s nasal cavity, including the folds in the mucous membrane, increases the surface area that captures scent molecules [63]. Combined with unique breathing patterns that allow for improved odor sampling, canines can detect even the faintest traces of VOCs. This biological advantage enables canines to differentiate and remember a wide array of smells, which is crucial for tasks involving VOC detection [64].

While the detection capacity of trained canines is impressive, inherent challenges and limitations have been associated with this method for clinical use, including variability in training-related performance, handler expertise, specific dog species, and animal ethics [65].

### 3.4. Use of Volatile Organic Compound Analysis for Detection of Cancer

VOC profiling offers a promising, non-invasive avenue for detecting metabolic alterations associated with malignancy. Between 2002 and 2022, VOC analyses for cancer detection were examined in 301 clinical trials. The most studied cancers included LC (n = 127), colorectal cancer (n = 27), head and neck cancer (n = 22), BC (n = 16), prostate cancer (n = 15), gastric cancer (n = 13), and bladder cancer (n = 10). Notably, only 38 studies included more than 100 cancer cases, the majority of which focused on LC [66]. Only a small number of studies reported analyzing urinary VOCs via GC-MS as biomarkers for diagnosing BC (5 studies, 26–65 cases, 21–70 controls) and LC (4 studies, 10–20 cases, 20–30 controls) [67].

The feasibility and accuracy of using VOC analysis for early detection of cancer, either by sensors (e-noses), GC modalities, or animals, has been reviewed by several groups. Goertzen et al. [68] reported a combined sensitivity and specificity above 150% across 44 studies primarily focused on cancer-related urine-based VOC analyses of prostate, lung, breast, and bladder cancer. Sensitivities ranging from 19–99% and specificities between 60–99% were reported for cancer detection by canines across multiple cancers, including lung, ovarian, prostate, colorectal, and bladder cancers, though these figures reflect limited sample sizes per cancer type [65]. In a comprehensive meta-analysis of studies evaluating cancer detection by VOC analysis of breath and/or urine, the overall specificity and sensitivity were 88% and 89%, respectively [69]. Hintzen et al. [70] reviewed VOC detection as a diagnostic tool for gastrointestinal cancers. Among 23 studies (14 on gastro-esophageal cancer and 2 on PDAC), sensitivity ranged from 66.7–100%, and specificity varied between 48.1% and 97.9%. Hanna et al. [71] reviewed 63 studies involving 3554 patients that examined breath-sampled VOCs for cancer detection, reporting a combined sensitivity of 79% (95% confidence interval [CI] 77–81%) and a specificity of 89% (95% CI 88–90%). A bio-AI hybrid system composed of trained canines and machine-learning models for detecting cancer-associated VOCs in breath samples demonstrated a combined sensitivity of 93.9% (95% CI 90.3–96.2%) and a specificity of 94.3% (95% CI 92.7–95.5%) in detecting BC, LC, colorectal, and prostate cancer, with an early-stage cancer (stage 0–2) sensitivity of 94.8%. Moreover, 81.8% sensitivity was noted for 14 additional cancer types that the canines were not trained to detect [72].

In summary, current evidence from reviews and meta-analyses indicates that VOC detection represents a promising non-invasive approach for complementing established cancer screening modalities. However, integration into clinical practice remains contingent upon addressing critical methodological limitations, including variability in sampling procedures, detection technologies, and validation strategies.

## 4. Using Artificial Intelligence in the Analysis of Volatile Organic Compounds and Liquid Biopsy

The increasing complexity and volume of biomedical data have positioned AI as a powerful means to extract clinically meaningful patterns, especially in contexts where the signal is subtle or noisy. AI-driven tools are particularly relevant for early cancer detection, where traditional diagnostic methods, such as imaging, biopsies, and molecular assays, often fall short due to invasiveness, limited sensitivity at early stages, or high costs [19,73,74].

Unlike conventional approaches that focus on a small set of predefined metabolites, AI enables analysis of the entire compositional “fingerprint” of VOCs, capturing subtle changes that may not be apparent through univariate or rule-based methods. Vinhas et al. [75] exemplified this emerging trend by using gas chromatography-field asymmetric Ion mobility spectrometry (GC-FAIMS) to analyze breath samples from LC patients and healthy individuals. The study involved training several neural network architectures, including GoogLeNet and VGG-11, on breath sample spectrograms. The models demonstrated strong classification performance, with precision and recall metrics indicating the potential of breathomics as a viable tool for early LC detection, particularly when augmented by AI. Other reviews [76,77] further underscore AI’s critical role in advancing breath-based diagnostics across a range of cancers, highlighting the advantages of machine learning over conventional statistical classifiers in handling the high-dimensional and often non-linear data that characterize VOC analyses.

In the same vein, AI has become indispensable in overcoming the limitations of interpreting LB data, particularly for early-stage cancers where biomarker abundance is low. For instance, Kim et al. [78] demonstrated that a deep-learning model applied to cfDNA methylation and fragment-size profiles can distinguish between patients with LC and healthy individuals with an accuracy of 81.5% and an area under the receiver-operating characteristic curve of 0.87. Yan et al. [79] showed that circulating tumor cell-derived RNA (ctcRNA)-based tissue deconvolution using a deep-learning approach can identify the tissue of origin of metastatic tumors.

Beyond single-analyte models, AI is increasingly used to integrate multiple biomarker types into unified diagnostic frameworks [80]. Medina et al. [81] utilized machine learning to analyze whole-genome cfDNA fragmentome, CA-125, and human epididymis protein 4, detecting OvC with specificity exceeding >99% and sensitivities of 72%, 69%, 87%, and 100% for stages I to IV, respectively. Such integrative approaches exemplify the strengths of AI in navigating multimodal data, especially in a clinical landscape moving toward personalized, biomarker-driven medicine.

## 5. Schemes and Methods for Early Detection of Specific Cancers

### 5.1. Breast Cancer

#### 5.1.1. Surveillance Schemes for Early Detection of Breast Cancer

The currently widely adopted screening scheme for early detection of BC in women at an average risk includes annual breast imaging, primarily by mammography, at ages 45–54 years. Starting at age 55 years, women can opt to either continue with annual mammograms or switch to a 2-year screening scheme [82,83]. This routine should be maintained for as long as the screened individual is in good health and expected to live at least 10 years. Recently, the USPSTF recommended that every average-risk woman aged 40–74 should obtain a mammogram every 2 years [8]. Notably, clinical breast exams, whether self-exams or physician-performed, have no clinically proven benefit in BC detection. For high-risk women, including those with *BRCA1* or *BRCA2* germline pathogenic variants, a history of breast irradiation during adolescence, or a 20% or higher lifetime risk for developing BC—determined by risk assessment algorithms such as BRCAPRO (https://projects.iq.harvard.edu/bayesmendel/brcapro accessed on 13 March 2025) or CanRisk (https://www.canrisk.org/ accessed on 13 March 2025)—it is recommended to begin BC screening at around age 30. The preferred imaging approach is magnetic resonance imaging (MRI), alternating with mammograms every 6 months [84].

The adoption of this screening methodology has been shown to positively impact early BC detection, improving survival rates and reducing the need for adjuvant chemotherapy [85]. However, this screening scheme is not without limitations. Challenges include overdiagnosis of slow-growing, non-invasive tumors (e.g., ductal carcinoma in situ), which may lead to overtreatment with surgery and irradiation; false-positive test results resulting in unnecessary biopsies and patient anxiety; and false-negative results leading to missed BC cases, particularly in young women and those with dense breasts. Additional concerns involve limited accessibility and fear of radiation exposure from mammography. To address these shortcomings, there is a need to enhance BC screening by incorporating additional methodologies. Noninvasive biomarkers, such as circulating cell-free tumor nucleic acids or microRNA, VOC detection in breath or urine, have been investigated as potential supplements to early-BC detection schemes [84].

#### 5.1.2. Liquid Biopsy Analysis for Breast Cancer Detection

Biomarker detection via LB has emerged as an effective approach for diagnosing BC, with peripheral blood microRNA analysis being the most extensively studied method [86]. However, small, early-stage tumors may go undetected using current LB schemes. For example, a multi-cancer cfDNA methylation assay (Galleri) detected only ~30% of new BC cases (only 2.6% of stage I cases) at ~99.5% specificity [87]. Similarly, another blood test (CancerSEEK) identified only one-third of early BC cases compared to 98% of OvC cases [88]. These results may be attributed to the low rate of tumor DNA shedding in early-stage BC. These caveats are addressed by combining multiple biomarker types (ctDNA, circulating proteins, CTCs), as well as by pairing LB with imaging to increase detection [89]. Large-scale trials, such as STRIVE [90] and PATHFINDER [31], are underway to refine this approach.

#### 5.1.3. VOC Analysis for Breast Cancer Detection

Several studies have examined VOC determination as a tool for early BC detection. Giró Benet et al. [91] reported 100% sensitivity but lower specificity (50–85.7%) in detecting BC in urine VOCs by an e-nose prototype (n = 90). A GC-based VOC analysis of breath samples from 54 women with BC and 124 cancer-free controls was associated with high negative and positive predictive values of 99.1% and 100%, respectively [92]. Using a high-throughput breathomics analysis by high-pressure photon ionization-time-of-flight mass spectrometry (HPPI-TOF-MS), Liu et al. [93] identified 10 optimal VOC markers to distinguish the breath samples of women with BC from those of women without cancer in a cohort of 5047 Chinese women undergoing BC screening. A diagnostic model (BreathBC-Plus) consisting of the 10 VOC markers and BC risk factors achieved detection rates of 96.97% for ductal carcinoma in situ, and 85.06%, 90.00%, 88.24%, and 100% for stages I, II, III, and IV BC, respectively. Phillips et al. [94] reported an accuracy of 83%, sensitivity of 82%, and specificity of 77.1% for BC detection in alveolar breath samples of 593 symptomatic women using a rapid point-of-care VOC detection method employing GC and surface acoustic wave detection. In another study, which examined a multi-omics approach combining metabolites in exhaled breath, ultrasound imaging, and basic clinical information, the breath samples of 1723 individuals were collected and analyzed using an automated breast volume scanner and HPPI-TOF-MS. This approach differentiated between benign (n = 168) and malignant (n = 82) breast masses, demonstrating sensitivity, specificity, and accuracy of 84.1% (95% CI 75.1–93.2%), 89.9% (95% CI 85.3–94.4%), and 88.0% (95% CI 84.0–92.0%), respectively [95].

A review of 32 studies examining various VOC detection methods using both in vivo and in vitro matrices for BC detection highlighted the potential of VOC analysis as a promising screening tool. However, the authors concluded that the findings are inconsistent, primarily due to methodological discrepancies [96]. Li et al. [84] also reviewed studies investigating noninvasive biomarkers, including VOCs, for BC detection. The studies, encompassing 10–54 BC cases and 10–543 controls, reported sensitivity rates ranging from 80–100% and specificity rates ranging from 40–77.1%.

### 5.2. Lung Cancer

#### 5.2.1. Surveillance Schemes for Early Detection of Lung Cancer

The American Cancer Society recommends annual LC screening using LDCT for individuals aged 50–80 years who are current or former smokers who have at least a 20 pack-year smoking history [97]. This recommendation is an adoption of the USPSTF statement issued in 2021 [98]. This recommended surveillance scheme is based on studies that have demonstrated a 20% decrease in LC mortality [99,100,101,102] and a 6.7% decrease in all-cause mortality [99], as well as several meta-analyses confirming the benefits of this screening scheme for reducing both disease-specific and all-cause mortality [103,104]. Yet, despite its proven contribution to reducing mortality rates, the adoption rate among individuals who may benefit the most from this screening scheme is low. According to the United Kingdom Lung Cancer Screen (UKLS) trial, the response rate to a lung screening invitation among those at the highest risk for LC was 6.1% [105]. Common reasons for not attending LDCT screening include difficulty accessing services, cancer fear, fatalism, negative psychological impact of screening, and stigma associated with LC and smoking [106,107,108,109]. In addition, several controversial and unresolved issues related to LDCT should be addressed to potentially increase participation rates among eligible individuals. These include false positive rates, over-diagnosis, costs, disparities among minority populations, radiation exposure, and incidental findings [110].

Thus, despite its proven effectiveness in reducing both disease-specific and overall mortality rates, LDCT remains underutilized among high-risk individuals. This underuse has driven the continued search for alternative methods to improve early LC detection.

#### 5.2.2. Liquid Biopsy Analysis for Lung Cancer Detection

Although lung tumors shed less DNA in early stages, ultrasensitive assays can detect tumor signals in many stage I patients [111]. For example, the multi-analyte blood test CancerSEEK demonstrated ~60% sensitivity for LC while maintaining ~99% specificity [88]. A cfDNA methylation assay from the Circulating Cell-Free Genome Atlas (CCGA) study achieved approximately 70% detection of LC across stages [112]. Although early detection of LC by LDCT has ~94% sensitivity [100], blood-based testing could complement imaging by using a two-step strategy: a reliable, valid LB could be used as an initial screening tool in high-risk smokers, identifying those who display an initial LC signal requiring a rapid and in-depth clinical analysis while reducing LDCT-based false positives [111]. Such a strategy may increase the overall detection rate and improve screening compliance. Ongoing studies, such as SUMMIT [113] and PATHFINDER [114], are currently evaluating the use of blood tests in real-world high-risk LC cohorts.

#### 5.2.3. VOC Analysis for Early Detection of Lung Cancer

Tsou et al. [115] used SIFT-MS to quantitatively analyze 116 VOCs in breath samples from 148 patients with histologically confirmed LC and 168 healthy volunteers. Using eXtreme Gradient Boosting (XGBoost), a machine-learning method, and adjusting for confounders such as environmental VOCs, they constructed a LC prediction model with an accuracy, sensitivity, and specificity of 92%, 96%, and 88%, respectively. Gasparri et al. [116] analyzed VOCs in exhaled breath using a gas sensor array to generate a “breathprint” of 70 individuals with LC and 76 non-cancer controls. They found that breathprints of individuals with LC were distinct from those of healthy individuals, with a sensitivity of 81% and specificity of 91%. The highest sensitivity was observed in individuals with stage I LC, highlighting the potential of VOC analysis for early detection. Kort et al. [117] explored non-invasive LC detection using an e-nose-based VOC detection integrated into a prediction model incorporating clinical parameters. Their study included 376 cases in the training set and 199 cases in the validation set. The prediction model showed a sensitivity of 95%, a specificity of 49%, and a negative predictive value (NPV) of 94% in the validation set.

Several reviews have reported on the use of various e-nose methods for LC detection [118,119,120,121] or for differentiating between benign pulmonary nodules and LC [122]. Across all studies, exhaled VOCs successfully distinguished between individuals with LC and controls. A meta-analysis, which examined VOCs in exhaled breath as biomarkers for LC screening, found an integrated sensitivity and specificity of 85% and 86%, respectively [123].

A few studies have explored the ability of canines to detect VOCs as a means for distinguishing LC from non-cancerous conditions. Buszewski et al. [124] found that trained dogs could differentiate between breath samples of individuals diagnosed with LC (n = 29, including 18 with small cell lung cancer and 11 with non-small cell lung cancer) from non-cancer controls (n = 44), achieving sensitivity and specificity of 82.2% and 82.4%, respectively. False positive rates in healthy controls were 17.8%. A positive correlation was observed between dog indications and the presence of ethyl acetate and 2-pentanone in exhaled breath (r  =  0.85 and r  =  0.97, respectively). McCulloch et al. [125] found that five household dogs demonstrated 99% specificity and sensitivity in distinguishing LC cases (n = 55) from non-cancer controls (n = 83), regardless of disease stage. Biehl et al. [126] further highlighted that factors such as dog training level, breed, and the VOC collection system significantly impact the accuracy of canine-based VOC detection of LC.

### 5.3. Pancreatic Cancer

PDAC remains an intractable condition, ranking among the leading causes of global cancer-related deaths [127]. Five-year survival rates range from only 3% to 15% when the disease extends beyond the pancreas [128,129]. This poor prognosis is primarily due to late-stage diagnosis, often when the disease is already incurable, as well as high tumor chemoresistance [130].

Currently, no effective early-detection schemes for PDAC have been proven to reduce mortality rates or improve survival in individuals at average risk [131]. Due to the rarity of PDAC and its nonspecific symptoms in early stages of the disease, it is generally recommended not to offer routine screening for individuals at average risk [131]. Some form of screening may be considered annually from age 50 for individuals who are at high risk for developing pancreatic cancer (e.g., those carrying inherited pathogenic variants in pancreatic cancer susceptibility genes—*BRCA2, PALB2, CDKN2A*; individuals with two or more first and/or second degree relatives from the same parental lineage diagnosed with PDAC; individuals with a history of pancreatitis, those with mucinous pancreatic cysts; and elderly patients with new-onset diabetes). For these high-risk groups, annual screening using magnetic resonance cholangiopancreatography combined with endoscopic ultrasound has been suggested. However, the clinical impact of such a screening is not yet well established, and further research is needed to determine its effectiveness [132,133]. Notably, *BRCA1* and *BRCA2* germline pathogenic variant carriers have a relatively low lifetime risk of PDAC, estimated at less than 5% [134].

#### 5.3.1. Liquid Biopsy for Pancreatic Cancer Detection

Since ctDNA is scarce in early-stage PDAC, LB aims to identify extracellular vesicles and microRNAs shed by pancreatic tumors [135,136,137]. Nakamura et al. [136] identified a panel of 15 exosomal microRNAs that, when combined with circulating free microRNAs, detected early-stage pancreatic cancer with an unprecedented 94% sensitivity and 97% specificity. The detection accuracy was further increased to ~97% for stage I–II disease when the classic CA19-9 protein marker was added in an independent cohort. While encouraging, these results need to be further independently validated. Other LB-based approaches, such as mutation-based ctDNA panels and methylation assays, are also being explored [138].

#### 5.3.2. VOC Analysis for Pancreatic Cancer Detection

Martínez–Moral et al. [139] identified 433 VOCs in serum samples by applying a GC-based technique, with 40 VOCs showing statistically significant differences between PDAC cases (n = 20) and controls (n = 19). Of these, 11 VOCs were independently validated, and 4 (toluene, 2-ethyl-1-hexanol, pentylbenzene, and butoxymethylbenzene) were identified as potential biomarkers. Daulton et al. [140] used two GC techniques to identify VOCs in urine samples in PDAC screening. This method effectively distinguished PDAC cases (n = 55) from healthy controls (n = 33) but failed to differentiate PDAC cases from those diagnosed with chronic pancreatitis (n = 45). Nissinen et al. [141] demonstrated 79% sensitivity and specificity in distinguishing PDAC cases (n = 68) from non-pancreatic cancer conditions (n = 62) and healthy controls (n = 52) using FAIMS for detecting urinary VOCs. Markar et al. [142] reported that VOC detection in breath samples by GC-MS identified 12 VOCs that could distinguish between PDAC cases (n = 57) and non-cancer cases (n = 75), with a sensitivity of 81% and a specificity of 58%. Princivalle et al. [143] reported that 10 VOCs detected in exhaled breath by an MS-based technique could distinguish between PDAC cases (n = 65) and non-cancer controls (n = 102) with a sensitivity and specificity of 100% and 84%, respectively. These results were subsequently validated in a smaller case-control cohort. Arasaradnam et al. [144] reported that VOC detection in urine samples by ion mobility spectrometry achieved 91% sensitivity (95% CI 0.83–0.96%) and 83% specificity (95% CI 0.73–0.90%) in detecting PDAC cases (n = 81) compared to controls (n = 81). Moreover, when comparing early-stage disease (stages I/II) to advanced-stage disease (stage III/IV), this approach showed a sensitivity of 82% (95% CI 0.67–0.92%) and a specificity of 89% (95% CI 0.75–0.97%).

### 5.4. Ovarian Cancer

#### 5.4.1. Ovarian Cancer Screening

OvC is a relatively rare cancer type, ranking as the 10^th^-most-common cancer among women. However, it is the fifth-leading cause of cancer-related deaths in females [13]. This high mortality rate is largely due to late-stage diagnosis, with nearly two-thirds of OvC cases diagnosed after the disease has already spread within the peritoneal cavity (stage III) or distant organs (stage IV) [145]. Indeed, the contrast in survival outcomes is stark: while the 5-year survival rate for localized OvC exceeds 90%, it drops to a mere 30% for metastatic disease [145].

Efforts at developing effective early-detection strategies for reducing OvC mortality have been ongoing since the 1980s [146]. Most large-scale studies have focused on serological markers, particularly CA-125, combined with ultrasonographic imaging of the ovaries and pelvic organs. While these methods have demonstrated the ability to detect OvC earlier and downstage the disease, they have consistently failed to show a significant reduction in mortality among women at average risk [147,148,149]. Notably, other screening modalities, including various serum markers and metabolic products, were studied over the years (e.g., ONCOVERYX-F [150]; CA-125:CA72-4 ratio [151]; reviewed by Davenport et al. [152]). However, none of these approaches have been validated as effective early-detection biomarkers, nor have they been widely adopted for clinical use.

Due to the lack of proven effect on mortality reduction and the relative rarity of OvC, many national health authorities advise against routine OvC screening for average-risk women [153,154]. For high-risk women, particularly those harboring germline pathogenic variants in OvC-susceptibility genes (e.g., *BRCA1, BRCA2, PALB2*), current recommendations emphasize active risk reduction through bilateral salpingo-oophorectomy at ages 35–45, depending on the specific mutated gene and family history, and after completing childbearing [153]. While this risk-reducing surgery significantly reduces OvC risk, it is associated with significant morbidity and negative effects on non-oncological health outcomes [155].

#### 5.4.2. Liquid Biopsy for Ovarian Cancer Detection

As no routine OvC screening is currently available for women with average and/or high risk, LB analyzing OvC-associated biomarkers, such as ctDNA, tumor-secreted microRNAs, and exosomal vesicles [38,41] may offer hope. One multi-omics approach combining cfDNA fragment patterns with protein markers (DELFI-Pro) outperformed traditional CA-125 testing. This test detected 72% of stage I and 87% of stage III OvC cases with >99% specificity, whereas CA-125 alone detected only 34% and 63% cases at these stages [81]. Importantly, an approximately 90% detection rate was noted for high-grade serous OvC, which is the most lethal subtype. Emerging strategies integrate AI algorithms and multi-marker panels to increase detection accuracy [156]. If confirmed in large independent cohorts, a cost-effective blood test for mass screening of OvC could become clinically available in the near future.

#### 5.4.3. VOCs for Ovarian Cancer Detection

In a study utilizing GC–MS and nanoarray-based VOC detection in breath samples of 48 women with OvC, 86 women with benign gynecological masses, and 48 healthy women, decanal, nonanal, styrene, 2-butanone, and hexadecane were identified as potential VOC markers for OvC. The sensitivity, specificity, and accuracy of this model were 79%, 100%, and 89%, respectively [128]. Raspagliesi et al. [157] demonstrated that an e-nose-based VOC analysis in breath samples collected from 86 women with OvC, 51 women with benign pelvic masses, and 114 controls, distinguished OvC cases from benign cases and controls with a sensitivity of 89% and specificity of 86%. In a proof-of-concept study involving four OvC cases, Horvath et al. [158] demonstrated the ability of canines to accurately detect OvC VOCs with a sensitivity of 100% and specificity of 97.5%. In a subsequent study by the same group, two trained dogs detected presymptomatic clinical recurrence of OvC in 42 women treated with chemotherapy with 97% sensitivity and 99% specificity [159]. Similarly, Kybert et al. [160] showed that three different dog breeds could distinguish among plasma and/or tissue samples from women with OvC (n = 10), women with benign gynecological conditions (n = 10), and plasma of healthy controls (n = 10). Detection rates varied between 93.3 ± 20.1% and 98.7 ± 5.1% depending on the breed.

Despite these promising findings, common limitations affecting VOC-based early detection of OvC include the small sample sizes, the variability in detection technologies and dog breeds, the lack of standardization across studies, and the failure to differentiate between sporadic OvC cases and those with an inherited predisposition, limiting the applicability of findings to high-risk populations.

## 6. Sex-Related Differences in Multicancer Early Detection for Pancreatic and Lung Cancer

Studies evaluating LB and VOC analyses for MCED have indicated minimal sex-related differences in detection rates for pancreatic and LC. LB technologies, such as the CancerSEEK [88] and Galleri [161] assays, reported no significant sex-based disparities in detection sensitivity. However, proteomics-based MCED approaches have shown subtle sex-specific performance variations. Budnik et al. [162] developed separate male- and female-optimized protein classifiers, achieving 90% sensitivity in men versus 85% in women at 99% specificity. VOC-based detection methods, such as the SpotitEarly bio-AI hybrid system [72], demonstrated equivalent high sensitivities (>90%) across both sexes for LC detection. Overall, while MCED technologies generally detect pancreatic and lung cancers effectively in both men and women, the most refined approaches are beginning to incorporate minor sex-based adjustments to further optimize accuracy.

## 7. Future Directions

Although current early-cancer-detection schemes are effective, they have inherent limitations that restrict their widespread adoption by the public. These challenges include targeting only a single cancer type, requiring specialized equipment and trained personnel, necessitating access to healthcare facilities, and, in some cases, involving exposure to ionizing radiation, albeit at low levels. Additionally, some prominent cancer types, such as PDAC and OvC, lack effective early-detection schemes, contributing to poor survival rates. Over the past 2 decades, basic and applied research has focused on developing non-invasive, accessible, easy-to-use, point-of-care, or at-home screening technologies that are also cost-effective. The ultimate goal is to establish an MCED approach that aligns with these criteria. Major clinical trials and studies utilizing these technologies are summarized in Table 1. Notably, several challenges must be considered when translating these technologies into clinical practice. Key limitations include the need for independent validation of findings in diverse populations with varying cancer prevalence, assessment of cost-effectiveness compared to developing alternatives (e.g., AI-assisted analyses of LDCT for LC and breast MRI for BC), and evaluation of assay scalability, accessibility, and regulatory compliance. Longitudinal studies are also essential to determine population-level impacts.

VOC analysis is a non-invasive approach showing high sensitivity and specificity, particularly in early-stage LC. It may improve screening uptake and reduce false positives from LDCT scans. In BC, VOCs demonstrate high sensitivity but variable specificity, making them promising as supplemental tools to mammography, especially for women with dense breast tissue or radiation concerns. VOCs can distinguish PDAC cases from healthy individuals with moderate-to-high sensitivity, but they struggle to differentiate from benign conditions like chronic pancreatitis. Breath- and canine-based VOC studies show high accuracy in detecting OvC in small cohorts, but clinical application is limited by a lack of standardization and small sample sizes. In contrast to VOC technologies, LBs are more established and increasingly tested in clinical trials, though early-stage sensitivity remains limited due to low biomarker shedding. In LC, LB complements imaging and may enhance early detection in high-risk groups. Early detection of breast tumors is challenging, but ongoing studies are exploring multi-marker and multi-omics strategies. In PDAC, combining exosomal microRNAs with markers like CA19-9 improves early-stage accuracy, though further validation is needed. For OvC, LB, especially multi-omics platforms, shows strong early-detection performance, particularly in high-grade serous cases, and could become a viable, non-invasive screening option if validated in larger studies.

An integrated multi-modal approach that combines diverse detection strategies holds significant potential for improving early cancer detection. One promising method is LB-based multi-omics analysis, which allows for simultaneous assessment of circulating genomic markers, proteomic signatures, and metabolomic profiles, together offering a comprehensive molecular fingerprint of cancer [163]. In fact, multi-analyte blood tests have already demonstrated the ability to detect multiple cancer types in asymptomatic individuals with high specificity [161,164]. VOC detection methods also show strong potential as a non-invasive complementary approach. Recent studies have reported high diagnostic accuracy using VOC analyses [58,164]. Increasingly, AI algorithms are being applied to these platforms, enabling the integration of complex multi-omics data and VOC signatures for improved pattern recognition and predictive power. Notably, animal-assisted detection, particularly using canine olfaction, underscores the potential of VOC-based diagnosis. Trained dogs have detected cancer via breath or urine samples with remarkable sensitivity, inspiring the development of AI-based bio-mimetic detection systems [72].

## 8. Conclusions

Overall, integrating multi-omics LB with VOC detection, augmented by advanced AI and even insights from canine olfaction, represents a promising frontier in non-invasive early cancer screening (Table 2). Such an integrated strategy offers the greatest potential for developing a standardized, clinically implementable system. Although we have not fully achieved this vision, we are undoubtedly progressing toward it.

## Figures and Tables

**Table 1:** Summary of studies evaluating VOC and liquid biopsy methods for cancer detection.

Study Name [References]	Cancer Type	Technology	Sample Size	Key Results
Schrag et al. [31] PATHFINDER	Multicancer	Liquid biopsy (Galleri methylation assay)	6621	Specificity: 99.1% Overall sensitivity: 66.3% Lower sensitivity for early stages
Cohen et al. [88] CancerSEEK	Multicancer	Liquid biopsy (ctDNA + protein markers)	1005	Specificity: ~99% Sensitivity across cancers: ~60–98%;
Liu et al. [93]	Breast cancer	VOC breath (HPPI-TOF-MS)	5047	Sensitivity across breast cancer stages: 85–97% High diagnostic accuracy
Kort et al. [117]	Lung cancer	VOC breath (Electronic nose)	575	Specificity: 49% Sensitivity: 95% Negative predictive value: 94%
Arasaradnam et al. [144]	Pancreatic cancer	VOC urine (Ion mobility spectrometry)	162	Specificity: 83% Sensitivity: 91%
Medina et al. [81] DELFI-Pro	Ovarian cancer	Liquid biopsy (cfDNA fragmentomics + protein markers)	591	Specificity: >99% Sensitivity: 72% (stage I), 69% (stage II), 87% (stage III), 100% (stage IV)
Half et al. [72] The Rainbow Study	Multicancer	VOC breath (Canine detection + AI)	1386	Specificity and sensitivity across cancers: 94%; ~82% for cancers that the system was not trained to detect

**Table 2:** Integrative strategy for enhanced cancer detection: combining canine VOC detection with liquid biopsy.

Cancer Detection Parameter	Liquid Biopsy Alone	Canine VOC Detection Alone	Integrated Approach
Early-stage Detection	Limited sensitivity for early-stage disease (24.2%) compared to later-stage (95.3%)	High sensitivity for early-stage cancers (94.8%)	Combined approach to capturing tumors missed by either method alone
Multi-cancer Detection	Effective for some cancer types, but variable performance across others	Can detect trained cancer types with high sensitivity (93.9%) and even untrained cancer types (81.8%)	Complementary detection across a wider range of cancer types
Specificity	High specificity (98.4–99.5%)	High specificity (94.3%)	Combined specificity could reduce false positives
Sample Requirements	Blood samples for cfDNA, ctDNA analysis	Breath, urine, or other bodily emissions	Multiple sample types enable cross-validation of results
Diagnostic Information	Provides genetic/molecular tumor profile for potential treatment guidance	Indicates presence of cancer but limited molecular details	Complete picture: detection plus molecular characterization
Integration with AI	AI algorithms enhance detection of subtle molecular patterns	Machine learning improves canine detection accuracy	AI platform integrating both data sources for superior pattern recognition

## Data Availability

Not applicable.

## Abbreviations

The following abbreviations are used in this manuscript:

AI	Artificial Intelligence
ASR	Age-Standardized Rate
BC	Breast Cancer
CfDNA	Circulating Free DNA
CTC	Circulating Tumor Cells
CtDNA	Circulating Tumor DNA
GC	Gas Chromatography
HPPI-TOF-MS	High-Pressure Photon Ionization-Time-of-Flight Mass Spectrometry
LB	Liquid Biopsy
LC	Lung Cancer
MCED	Multi-Cancer Early Detection
MS	Mass Spectrometry
NCCN	National Comprehensive Cancer Network
OvC	Ovarian Cancer
PDAC	Pancreatic Ductal Adenocarcinoma
SIFT-MS	Selected Ion Flow Tube Mass Spectrometry
USPSTF	US Preventive Services Task Force
VOC	Volatile Organic Compounds
